# Glucagon-like peptide-1 receptor agonists and the endothelium: molecular and clinical insights into cardiovascular protection

**DOI:** 10.3389/fmed.2025.1669685

**Published:** 2025-09-09

**Authors:** Allegra Battistoni, Linda Piras, Nicola Tartaglia, Francesco Maria Carrano, Claudia De Vitis, Emanuele Barbato

**Affiliations:** ^1^Department of Clinical and Molecular Medicine, Sapienza University of Rome, Rome, Italy; ^2^Department of Medical and Surgical Sciences and Translational Medicine, Sapienza University of Rome, Rome, Italy

**Keywords:** obesity, diabetes, cardiovascular disease, endothelial dysfunction, incretins, GLP1, GLP1-RA

## Abstract

Endothelial dysfunction represents the critical pathophysiological mediator linking the modern epidemics of obesity, type 2 diabetes mellitus, and cardiovascular disease. Persistent hyperglycemia and metabolic dysregulation promote oxidative stress, reduce nitric oxide bioavailability, and activate inflammatory pathways, thereby accelerating atherosclerosis and cardiovascular complications. Therefore, strategies aimed at restoring endothelial function are crucial to mitigate cardiovascular complications in individuals with cardiometabolic disorders. Among antidiabetic therapies, glucagon-like peptide-1 receptor agonists have demonstrated cardiovascular benefits in large-scale outcome trials, but the underlying mechanisms remain only partially elucidated. In this mini-review, we critically examine both clinical and experimental evidence, with emphasis on the direct effects of glucagon-like peptide-1 receptor agonists on endothelial function. Moreover, we address the heterogeneity within this drug class, noting how differences may contribute to variability in vascular outcomes. By integrating clinical findings with molecular data, this review aims to refine our understanding of the potential endothelial mechanisms underlying cardiovascular protection. Our critical synthesis provides a clearer framework for interpreting the vascular effects of glucagon-like peptide-1 receptor agonists beyond glycemic control, thereby offering a more comprehensive view of their role in managing cardiometabolic disease.

## 1 Introduction

Cardiovascular diseases (CVD), type 2 diabetes mellitus (T2DM), and obesity constitute a convergent triad representing a major global health burden. Diabetes affects an estimated 463 million individuals worldwide, a number that has quadrupled since 1980 (1). More than half of these diabetes-related deaths are attributable to macrovascular complications, underscoring CVD as the principal driver of mortality in T2DM (2). This alarming overlap is not coincidental but rather reflects shared pathophysiological mechanisms, chief among them, endothelial dysfunction (ED). The vascular endothelium plays a pivotal role in maintaining vascular tone, regulating fibrinolytic and thrombotic activity, and preventing leukocyte and platelet adhesion (3). When compromised, the endothelium fosters a pro-inflammatory and pro-atherogenic environment, leading to impaired vasodilation and accelerated atherosclerosis (4, 5). ED is widely considered an early hallmark of CVD and is particularly prevalent in individuals with insulin resistance, hyperglycemia, and T2DM (6). In parallel, obesity has reached pandemic proportions, affecting close to one billion people worldwide (7). It is not only a primary driver of T2DM, but also an independent contributor to CVD risk (2–8). For each unit increase in BMI, the risk of developing diabetes and cardiovascular complications rises significantly (9). Because ED biologically links type 2 diabetes, obesity, and CVD, therapies capable of acting across all three pathways are urgently needed. In this context, glucagon-like peptide-1 receptor agonists (GLP-1 RAs) have emerged not only as effective glucose-lowering and weight-reducing agents but also as promising modulators of cardiovascular and endothelial health (10–12). The present mini-review therefore aims to summarize the contribution of ED to CVD in obesity and T2DM, to synthesize the pre-clinical and clinical evidence supporting endothelial and cardiovascular benefits of GLP-1RAs, and to delineate current controversies and research priorities that will inform future incretin-modulating interventions.

## 2 Incretins, GLP-1 receptors, and therapeutic agonists

Incretins are a class of gut-derived peptide hormones that enhance insulin secretion in response to nutrient ingestion, playing a key role in postprandial glucose regulation. The two primary incretins identified in humans are glucose-dependent insulinotropic polypeptide and glucagon-like peptide-1 (GLP-1). Both act through specific G protein–coupled receptors predominantly expressed on pancreatic β-cells, where they stimulate insulin release in a glucose-dependent manner, thus minimizing hypoglycemia risk (13). Beyond their islet effects, incretins exert pleiotropic actions on the brain (appetite and satiety regulation), gastrointestinal tract (delayed gastric emptying), adipose tissue (lipolysis modulation), and liver (inhibition of hepatic glucose output) (13). These wide-ranging actions reflect the evolutionary importance of incretin signaling in nutrient metabolism and energy homeostasis. GLP-1, in particular, is secreted by enteroendocrine L-cells of the distal ileum and colon following food intake. It exists mainly as GLP-1 (7–36) amide and GLP-1 (7–37), both of which are rapidly degraded by the enzyme dipeptidyl peptidase-4 (DPP-4) into the inactive metabolite GLP-1 (9–36), giving the active form a plasma half-life of only 1–2 min (14, 15). GLP-1 exerts its biological effects via the GLP-1 receptor (GLP-1R), a class B heptahelical G protein-coupled receptor coupled to Gs proteins that stimulate adenylyl cyclase and increase intracellular cyclic adenosine monophosphate (cAMP) (16). While the receptor is abundantly expressed in pancreatic β-cells, it is also present in cardiovascular tissues (heart, vasculature), kidneys, lungs, and central nervous system (17–18–19). However, the exact localization and functional significance of GLP-1R in the cardiovascular system are still being unraveled (16). The pharmacologic exploitation of GLP-1 signaling has led to two major therapeutic strategies: DPP-4 inhibitors, which prolong endogenous GLP-1 activity, and GLP-1 RAs, which mimic or enhance GLP-1 action while resisting enzymatic degradation (13). GLP-1 RAs are administered via injection and can be categorized by duration of action into short-acting (e.g., exenatide BID, lixisenatide) and long-acting forms (e.g., liraglutide, semaglutide, dulaglutide). These agents are derived either from human GLP-1 analogs or non-mammalian sequences such as exendin-4 (13). In addition to glycemic control, GLP-1 RAs induce significant weight loss, establishing them as foundational therapies in the management of T2DM and obesity (17).

## 3 Cardiovascular effects of GLP-1 receptor agonists: clinical evidence beyond glycemic control

Since the FDA mandate in 2008 to demonstrate cardiovascular safety for new glucose-lowering agents, a series of large randomized cardiovascular outcome trials have evaluated GLP-1 RAs in patients with T2DM, many of whom had established atherosclerotic cardiovascular disease (ASCVD) or multiple risk factors. These trials, while originally designed to establish non-inferiority, have consistently revealed a broader cardioprotective signal in several agents, suggesting that the benefits extend beyond glucose control. The LEADER trial enrolled patients with T2DM and high cardiovascular risk, defined as established CVD (81%) or age ≥60 years with at least one risk factor, and showed that liraglutide significantly reduced the incidence of 3-point major adverse cardiovascular events (MACE) (i.e., CV death, non-fatal myocardial infarction, non-fatal stroke) by 13% compared to placebo (HR 0.87; *p* = 0.01), along with a reduction in all-cause mortality (HR 0.85; *p* = 0.02) over a median follow-up of 3.8 years (18). Similarly, the SUSTAIN-6 trial, which enrolled patients with T2DM and either established CVD, stage ≥3 chronic kidney disease, or age ≥60 with risk factors, showed that semaglutide reduced MACE by 26% (HR 0.74), primarily driven by a 39% reduction in non-fatal stroke (19). Albiglutide in the Harmony Outcomes trial showed a 22% reduction in MACE in patients with established ASCVD (20), while exenatide (EXSCEL trial) (21) and lixisenatide (ELIXA trial) (22) did not meet superiority, though both demonstrated CV safety. Notably, the EXSCEL trial had a high treatment discontinuation rate (~43%), potentially diluting its results. In the PIONEER 6 trial, involving patients with T2D and high CV risk (including established CVD, chronic kidney disease, or age ≥50 years with risk factors), oral semaglutide demonstrated non-inferiority for MACE (HR 0.79; 95% CI, 0.57–1.11), with significant reductions in CV death (HR 0.49) and all-cause mortality (HR 0.51), though the trial was not powered to demonstrate superiority (23). The REWIND trial, which notably included a broader population-only 31% of participants had established CVD-showed that dulaglutide still reduced MACE by 12% (HR 0.88; *p* = 0.026), suggesting potential benefit even in primary prevention contexts (24). Finally, the AMPLITUDE-O trial, which included patients with T2D and either a history of CVD (90%) or CKD, demonstrated that efpeglenatide significantly reduced MACE by 27% (HR 0.73; *p* ##### 0.001), confirming the class effect even with agents structurally distinct from native GLP-1 (25). Overall, meta-analyses suggest a class effect in reducing MACE, particularly with long-acting agents (26). Importantly, the magnitude and consistency of MACE reduction in several trials exceed what would be expected from glycemic or weight improvements alone. Blood pressure, lipid profile, and body weight all improved modestly, but not sufficiently to explain the full cardiovascular benefit. This suggests a possible direct action of GLP-1 RAs on the cardiovascular system (27). Recent trials in heart failure (HF) have further clarified the therapeutic potential of GLP-1 RAs beyond glycemic control. While earlier trials in HFrEF (28, 29) were neutral or inconclusive, the STEP-HFpEF and SELECT trials highlighted robust improvements in patients with heart failure with preserved ejection fraction (HFpEF), particularly in obese phenotypes. In STEP-HFpEF, semaglutide led to improvements in symptoms, physical function, and quality of life metrics, with associated reductions in body weight and inflammation (30). Similarly, in the SUMMIT trial, tirzepatide significantly reduced a composite of death and HF worsening in obese HFpEF patients (HR 0.41–0.67), along with measurable improvements in functional status and exercise tolerance (31). Most strikingly, the SELECT trial demonstrated that semaglutide significantly reduced MACE in individuals without T2DM but with overweight or obesity and established CV risk (HR 0.80), making it the first GLP-1 RA approved for CV risk reduction in a non-diabetic population (32). This is a critical proof-of-concept that GLP-1 RAs effects are not mediated solely through glycemic control but may involve direct modulation of vascular inflammation, endothelial dysfunction, and myocardial energetics. Taken together, these findings establish GLP-1 RAs as agents with multidimensional benefits.

## 4 Endothelial dysfunction as a central mechanism in cardiovascular disease: molecular insights

ED plays a central role in the pathogenesis and progression of CVD, including atherosclerosis, hypertension, HF, stroke, and peripheral artery disease. The endothelium is not merely a passive barrier lining blood vessels but a highly dynamic and heterogenous organ with autocrine, paracrine, and endocrine functions. It regulates vascular tone, blood flow, hemostasis, inflammation, and angiogenesis (33, 34). One of the hallmark features of ED is reduced nitric oxide (NO) bioavailability. NO is a vasodilator synthesized by endothelial nitric oxide synthase (eNOS) in response to shear stress and stimuli such as acetylcholine. It inhibits vascular smooth muscle cell (VSMC) proliferation, platelet aggregation, and leukocyte adhesion (35). Impaired NO synthesis or increased NO degradation due to oxidative stress reduces vasodilatory capacity, promotes vasoconstriction, and enhances vascular tone, contributing to hypertension and ischemia (36, 37). Reduced NO levels also induce the expression of adhesion molecules, such as intercellular adhesion molecule-1 (ICAM-1), vascular cell adhesion molecule-1 (VCAM-1), and E-selectin, which mediate leukocyte recruitment to sites of endothelial injury (38). These leukocytes internalize oxidized low-density lipoprotein (oxLDL), promoting foam cell formation and early atherogenesis. Endothelial activation, marked by increased permeability and leukocyte adhesion, further fuels vascular inflammation and plaque progression (39). Oxidative stress is a key driver of ED. Reactive oxygen species (ROS), generated by nicotinamide-adenine-dinucleotide-phosphate (NADPH) oxidases, mitochondrial dysfunction, and uncoupled eNOS, deplete NO and oxidize cellular lipids and proteins (40). Exogenous factors such as hyperglycemia, smoking, and dyslipidemia exacerbate ROS production (41). ROS compromise tight junction integrity, increase endothelial permeability, and permit inflammatory cell infiltration, amplifying vascular damage. In a translational context, targeting ROS, through NADPH oxidase inhibitors or mitochondrial antioxidants, have shown promise in restoring endothelial function, with emerging biomarkers of oxidative stress offering potential for clinical monitoring and therapeutic stratification (42). Persistent oxidative stress creates a self-perpetuating cycle of endothelial injury, inflammation, and vascular remodeling (33). Inflammation is both a cause and consequence of ED. Pro-inflammatory cytokines like tumor necrosis factor-alpha (TNF-α), interleukin-1β (IL-1β), and interleukin-6 (IL-6), along with chemokines such as monocyte chemoattractant protein-1 (MCP-1), are upregulated in ED and drive immune cell recruitment and cytokine release (39–43). Chronic low-grade inflammation, as seen in diabetes, hypertension, and metabolic syndrome, initiates and perpetuates ED, ultimately leading to plaque destabilization and thrombosis. A more recently recognized contributor to ED is the endothelial-to-mesenchymal transition, a process by which endothelial cells acquire mesenchymal characteristics, losing markers such as VE-cadherin and gaining fibrotic and migratory phenotypes (47–37). Endothelial-to-mesenchymal transition is induced by high glucose, TGF-β signaling, and inflammatory cytokines, and is implicated in fibrosis and plaque instability (33, 34). In conclusion, ED is not merely a marker but a mechanistic driver of CVD. It integrates hemodynamic, metabolic, and inflammatory insults through molecular pathways centered around NO deficiency, oxidative stress, and immune activation. Several therapeutic strategies aim to restore endothelial function, with growing evidence supporting GLP-1RAs.

## 5 Endothelial-protective effects of GLP-1 receptor agonists: mechanistic insights from preclinical and clinical evidence

**Figure 1:**
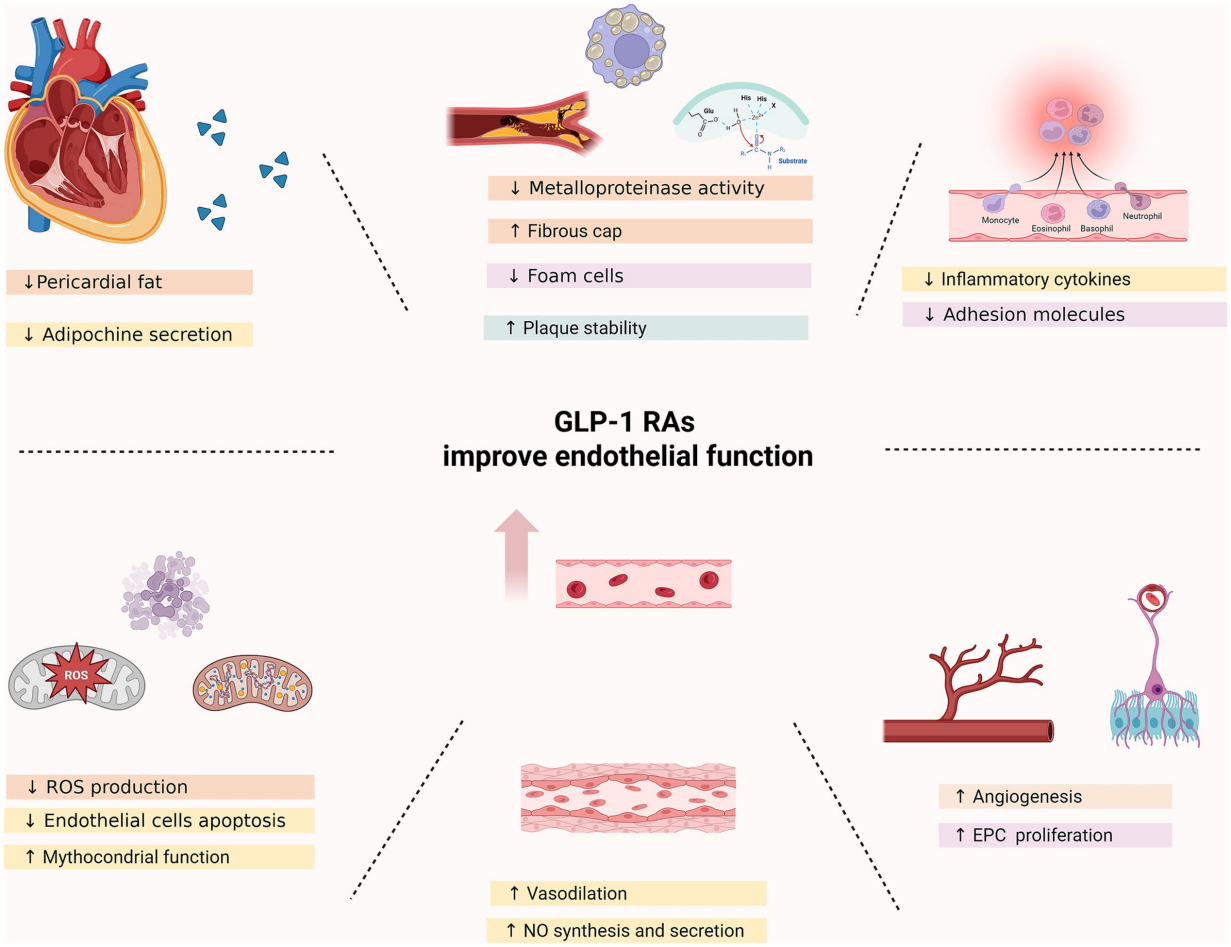
Effects of GLP1-RA on endothelium.

GLP-1RAs exert profound protective effects on the vascular endothelium, impacting key pathways involved in endothelial homeostasis, inflammation, oxidative stress, and vascular regeneration. These pleiotropic effects are increasingly recognized as central to the cardiovascular benefits observed in major clinical trials of GLP-1RAs. Below, we synthesize current evidence from both experimental and clinical studies elucidating how GLP-1R activation promotes endothelial health and counteracts atherogenesis (Figure 1).

### 5.1 Promotion of angiogenesis and endothelial progenitor cell function

Endothelial progenitor cells (EPCs) play a pivotal role in endothelial repair and post-ischemic angiogenesis. These cells, derived from bone marrow, can differentiate into mature endothelial cells, contributing to vascular regeneration, particularly in response to injury or ischemia. Their number and function are highly sensitive to oxidative stress, glycation end products, and inflammation (44). GLP-1RAs enhance EPC number and function through multiple mechanisms. Clinical studies in patients with T2DM have shown that treatment with dulaglutide increases circulating EPCs and boosts their proliferative, adhesive, migratory, and tubulogenic capacity (45, 46). This improvement correlates with reductions in pro-inflammatory markers such as IL-6, TNF-α, C-reactive protein, and advanced glycation end-products, supporting a role for GLP-1RAs in modulating EPC function through anti-inflammatory and antioxidative pathways (45, 46). Furthermore, high glucose reduces GLP-1R expression in EPCs, impairing their function and promoting apoptosis. Restoration of GLP-1 signaling using exendin-4 reverses these effects, highlighting the direct role of GLP-1R in EPC biology (44, 47). Moreover, GLP-1RAs stimulate endothelial VEGF expression, a fundamental trigger for angiogenesis, leading to activation of downstream VEGF receptors (VEGFR-2), which initiate angiogenic cascades involving phospholipase C gamma/extracellular signal-regulated kinase/phosphoinositide 3-kinase/protein kinase B pathways. This molecular signaling enhances EC proliferation, migration, and tubulogenesis. In primary human umbilical vein endothelial cells (HUVECs) treated with GLP-1RAs, increased VEGF production is accompanied by improved proliferation and tube formation, reflecting restored angiogenic capacity (44).

### 5.2 Reduction of oxidative stress and endoplasmic reticulum stress

Chronic hyperglycemia and endothelial activation drive excessive generation of ROS, promoting mitochondrial dysfunction, endothelial apoptosis, and reduced NO bioavailability. GLP-1RAs mitigate these effects through a combination of antioxidant, anti-apoptotic, and mitochondrial-stabilizing actions. Several studies report that GLP-1RAs reduce ROS production, recover mitochondrial membrane potential, and improve oxygen consumption in endothelial cells. These effects are accompanied by increased leukocyte rolling velocity and reduced leukocyte adhesion-indicators of improved endothelial barrier function (45). At the molecular level, exendin-4 activates adenosine monophosphate-activated protein kinase (AMPK) and upregulates endoplasmic reticulum oxidoreductin 1 alpha, which enhances the protein-folding machinery in endothelial cells, suppressing ER stress and ROS overproduction (48). In hyperhomocysteinemia models, this mechanism restores endothelial function and suppresses oxidative damage. Moreover, GLP-1RAs inhibit NADPH oxidase (NOX4)-dependent ROS production and downregulate NLRP3 inflammasome activation, both critical mediators of endothelial injury in diabetes (49). Liraglutide has also been shown to reduce TRIB3/nuclear factor kappa-light-chain-enhancer of activated B cells (NF-κB)/inhibitor of κB alpha signaling, thereby attenuating oxidative stress and pyroptosis in HUVECs (50). Translational studies demonstrate that GLP-1RAs, such as liraglutide, restore endothelial redox balance by reducing NOX4-derived ROS and enhancing mitochondrial function, effects corroborated in both HUVECs and diabetic animal models of vascular dysfunction. These findings support their emerging role in preserving endothelial integrity and preventing atherosclerotic progression beyond glycemic control (45).

### 5.3 Improvement of vasodilation and NO bioavailability

A defining feature of GLP-1RA-mediated endothelial protection is the restoration of endothelial-dependent vasodilation. Clinical and preclinical data consistently demonstrate enhanced NO production and eNOS phosphorylation following GLP-1RA treatment (44–51). In ApoE^−/−^ mice, liraglutide increased endothelium-derived NO as evidenced by enhanced contractile response to Nitro-L-Arginine Methyl Ester, confirming improved NO availability (43). Parallel *in vitro* studies showed increased eNOS and expression mediated by AMPK and Phosphoinositide 3-Kinase / Protein Kinase B signaling pathways, implicating a GLP-1R/cAMP/protein kinase A (PKA) axis in the upregulation of endothelial NO production (51). These effects are functionally significant: GLP-1RAs improve coronary and brachial artery flow, as shown in both healthy individuals and patients with diabetes, independent of glucose control (51). Notably, exenatide also enhances ATP-sensitive potassium channel activity, contributing to improved vasomotor responsiveness following ischemia-reperfusion injury (52).

### 5.4 Anti-inflammatory effects and endothelial adhesion molecule regulation

GLP-1RAs exert potent anti-inflammatory effects on the endothelium and immune cell interfaces, inhibiting key processes in leukocyte recruitment and adhesion. A hallmark of atherosclerosis is endothelial expression of adhesion molecules such as ICAM-1, VCAM-1, and E-selectin, which mediate monocyte recruitment. GLP-1RAs reduce the expression of these molecules *in vitro* and *in vivo*. For instance, liraglutide suppresses TNF-α-induced ICAM-1 and VCAM-1 expression in endothelial cells, an effect mediated via NF-κB inhibition (14–43, 53–55). Similarly, exenatide reduces soluble ICAM-1 and VCAM-1 in patients with type 2 diabetes, indicating systemic anti-inflammatory action (51). Additionally, semaglutide alters the secretory profile of epicardial adipose tissue, increasing its anti-thrombotic and anti-inflammatory properties by downregulating FABP4 expression (56). In vascular macrophages, exendin-4 suppresses lipopolysaccharide-induced pro-inflammatory gene expression by activating the cAMP/PKA pathway and inhibiting NF-κB nuclear translocation, further supporting a systemic immunomodulatory role (57).

### 5.5 Inhibition of atherosclerotic plaque formation and stabilization

Collectively, the endothelial and immunological actions of GLP-1RAs culminate in reduced atherogenesis and enhanced plaque stability. Preclinical models have demonstrated that GLP-1RAs reduce plaque area, monocyte/macrophage accumulation, and plaque vulnerability (37–48–49). For example, treatment with semaglutide reverses Western diet-induced aortic gene expression patterns related to leukocyte trafficking, lipid metabolism, and extracellular matrix turnover-key contributors to atherogenesis (58). Moreover, GLP-1RA therapy leads to plaques characterized by reduced inflammation and increased stability due to suppression of matrix metalloproteinases and promotion of fibrous cap formation (44, 45). Human data further support these findings: GLP-1RA treatment is associated with reduced carotid intima-media thickness and lower circulating inflammatory biomarkers, suggesting tangible antiatherogenic effects in patients (45, 46, 48–52, 56–59). Finally, beyond their effect on the vascular wall, GLP-1RAs may reduce thrombosis risk by inhibiting platelet aggregation, although the precise mechanisms-whether endothelial-dependent or independent-remain under investigation (35).

### 5.6 Effects of GLP-1 receptor agonists on endothelial function *in vivo*

Flow-mediated dilation (FMD) is a well-established, noninvasive, endothelium-dependent method for assessing vascular function. It measures the change in brachial artery diameter in response to ischemia via ultrasound and reflects vascular elasticity and endothelial integrity (60). Systematic reviews and meta-analyses have explored the impact of antidiabetic agents on vascular function, showing that GLP-1RAs significantly improve FMD compared to placebo (61, 62). Notably, GLP-1RAs demonstrated superior improvements in FMD compared to sulfonylureas and lifestyle interventions in multiple randomized controlled trials, including in patients with T2DM without overt CVD. Network meta-analyses have confirmed these effects with robust consistency (*I*^2^ = 0%) and no significant heterogeneity (61). However, some individual studies reported variable results (63). Conversely, treatment with exenatide LAR was associated with improved FMD and carotid intima-media thickness, alongside metabolic benefits, in patients with type 2 diabetes (64).

## 6 Discussion

The global rise in obesity and T2DM has led to a parallel pandemic of CVD, forming a cardio-metabolic triad that now represents a leading cause of morbidity and mortality worldwide (65). At the intersection of these conditions lies the endothelium, a dynamic regulator of vascular homeostasis, whose dysfunction initiates and accelerates atherosclerosis and associated complications. GLP-1RAs first emerged as glucose-lowering drugs, but recent findings highlight their multifactorial role in endothelial protection, particularly in the context of metabolic and atherosclerotic disease (44). Rather than acting through a single pathway, these agents exert coordinated effects across several levels of vascular regulation. This pleiotropic profile suggests that GLP-1RAs may go beyond glycemic control to provide direct vascular benefits, a hypothesis increasingly supported by both preclinical and clinical data. Among the most relevant effects, the ability of GLP-1RAs to restore EPC number and function deserves particular attention, especially considering the impairment of EPCs in diabetes and their central role in endothelial repair (14–37– 50). By promoting VEGF and NO signaling, these agents enhance angiogenic potential that may translate into improved microvascular integrity. Similarly, the reduction of ROS and restoration of mitochondrial function (14) position GLP-1RAs as valuable tools against oxidative stress-driven endothelial injury. Lastly GLP1-RA proved to reduce inflammation (14–49–54–57). These effects might represent the potential mechanisms underlying the reduction in cardiovascular events observed in some clinical trials. Collectively, these trials reinforce that the cardiovascular benefits of GLP-1RAs are not restricted to one molecule or only injectable formulations, and may extend to lower-risk populations. Despite the consistency of MACE reduction across several agents, different trial designs, population, and exposure, argues for a true class effect, yet several critical issues remain unresolved. First, the heterogeneity among GLP-1RAs, particularly regarding their molecular structure and pharmacokinetics, may account for divergent cardiovascular outcomes. Human GLP-1 analogs such as liraglutide and semaglutide demonstrate more consistent cardioprotection compared to exendin-4-based agents like exenatide, which may suffer from lower receptor affinity, greater immunogenicity, and less favorable pharmacokinetic profiles (49). These differences might partly explain the neutral results of the EXSCEL trial with exenatide (22). Second, the translational relevance of *in vitro* studies remains uncertain. Many experiments utilize supraphysiological concentrations of GLP-1RAs, potentially engaging non-canonical receptors or producing artifacts not reflective of clinical scenarios (34). This calls for a more cautious interpretation of molecular findings and underlines the need for dose–response studies *in vivo*. Lastly, while current CVOTs have provided encouraging data, they have largely focused on patients with T2DM. The ongoing SURPASS-CVOT and SYNCHRONIZE-CVOT trials will expand this landscape, testing the cardiovascular efficacy of dual agonists like tirzepatide and survodutide in broader populations, including those with obesity and established cardiovascular risk (ClinicalTrials.gov identifiers: NCT04255433, NCT05556512). These studies may help clarify whether the endothelial benefits observed preclinically translate into meaningful clinical outcomes across diverse patient groups. In summary, GLP-1RAs exhibit substantial potential in reversing ED via multiple integrated mechanisms. However, further investigation is warranted to clarify drug-specific actions, confirm the translational relevance of preclinical findings, and evaluate long-term vascular outcomes in broader patient populations, including those without T2DM. Comparative studies and real-world data will be crucial to better define their cardiovascular impact. Future research should also explore potential synergies with other antidiabetic agents and assess effects on validated clinical vascular biomarkers such FMD. Notably, despite the central role of ED in the pathogenesis of CVD, its clinical application as a prognostic or diagnostic tool remains limited. Various biomarkers and functional assessments of endothelial health, have demonstrated predictive value in research settings; however, their integration into routine clinical practice for cardiovascular risk stratification is still lacking. Bridging this gap between mechanistic insight and clinical utility represents a critical challenge for future translational research.
